# Targeted transcriptional and proteomic studies explicate specific roles of *Bacillus subtilis* iturin A, fengycin, and surfactin on elicitation of defensive systems in mandarin fruit during stress

**DOI:** 10.1371/journal.pone.0217202

**Published:** 2019-05-23

**Authors:** Paiboon Tunsagool, Wichitra Leelasuphakul, Janthima Jaresitthikunchai, Narumon Phaonakrop, Sittiruk Roytrakul, Warangkana Jutidamrongphan

**Affiliations:** 1 Department of Biochemistry, Faculty of Science, Prince of Songkla University, Songkhla, Thailand; 2 Proteomics Research Laboratory, National Center for Genetic Engineering and Biotechnology, National Science and Technology Development Agency, Pathum Thani, Thailand; 3 Faculty of Environmental Management, Prince of Songkla University, Songkhla, Thailand; Texas A&M University System, UNITED STATES

## Abstract

Application of *Bacillus* cyclic lipopeptides (CLPs); fengycin, iturin A and surfactin has shown a great potential in controlling the spread of green mold pathogen invasion (*Penicillium digitatum*) in wounded mandarin fruit during postharvest period. The limited defensive protein profiles followed specific expression of pivotal genes relating to plant hormone mediating signaling pathways of the CLPs’ action on stimulating host plant resistance have been exhibited. The present study aimed to elucidate the specific effect of individual CLP obtained from *Bacillus subtilis* ABS-S14 as elicitor role on activation of plant defensive system at transcriptional and proteomic levels with and without *P*. *digitatum* co-application in mandarin fruit. Fengycin and iturin A elevated the gene expression of *PAL*, *ACS1*, *ACO*, *CHI*, and *GLU* while significantly stimulating plant *POD* transcription was only detected in the treatments of surfactin both with and without following *P*. *digitatum*. An increase of *LOX* and *PR1* gene transcripts was determined in the treatments of individual CLP with fungal pathogen co-application. Fengycin activated production of unique defensive proteins such as protein involved in ubiquinone biosynthetic process in treated flavedo without *P*. *digitatum* infection. Proteins involved in the auxin modulating pathway were present in the iturin A and surfactin treatments. CLP-protein binding assay following proteome analysis reveals that iturin A attached to 12-oxophytodienoate reductase 2 involved in the oxylipin biosynthetic process required for jasmonic acid production which is implicated in induced systemic resistance (ISR). This study suggests specific elicitor action of individual CLP, particularly iturin A showed the most powerful in stimulating the ISR system in response to stresses in postharvest mandarins.

## Introduction

Fruit decay caused by *Penicillium digitatum* is one of the main problems of economic losses of the citrus crops often occurs during postharvest handling. The application of fungicides after harvesting has been shown to be the most effective control method for reduction of postharvest loss caused by the fungal diseases. Nevertheless, the continuous use of the certain chemical fungicides in long term of postharvest crops has led to the fungal pathogenic development of resistant strains against the certain chemical fungicides [1]. Subsequently, the biological control of postharvest fruit quality by means of natural plant-associated microorganisms such as *Bacillus* spp. has been introduced to be a safe and well-established crop productivity management technique which eliminates the devastating environmental and health impacts of the use of harmful chemical pesticides. The antimicrobial effect of *Bacillus subtilis* based on the production of secondary metabolites such as hydrolytic enzymes and volatile organic compounds. *B*. *subtilis* produces cyclic lipopeptides (CLPs; iturin A, fengycin and surfactin), and encourages citrus fruit to act against *P*. *digitatum*, a green mold rot pathogen, as reported in a previous study [2]. The involvement of *Bacillus* CLPs has been demonstrated in antimicrobial activity against various fungal and bacterial pathogens and they are also known for their elicitor role in stimulating plant-host-induced systemic resistance (ISR) [3]. Jasmonic acid (JA) and ethylene (ET) are the pivotal regulators in ISR [4]. Lipoxygenase (LOX) is an important enzyme in JA biosynthesis [5] while 1-aminocyclopropane-1-carboxylic acid synthase (ACS) and 1-aminocyclopropane-1-carboxylic acid oxidase (ACO) are the key enzymes in ET biosynthesis [6]. Moreover, systemic acquired resistance (SAR) is a plant defensive mechanism which depends on the salicylic acid (SA) signaling pathway and phenylalanine ammonia lyase (PAL) is an important enzyme in synthesizing SA accumulation [7].

Antifungal activity of CLPs generated by *Bacillus* species was reported to function in antibiosis through sterol and phospholipid molecules which stimulate pore formation, thus affecting the permeability of the fungal cell membrane [8]. Moreover, the CLPs comprising, iturin A, fengycin and surfactin, have also been found to induce defensive gene transcription for plant resistance [3, 9]. Fengycin isolated from the antagonistic *B*. *subtilis* strain ABS-S14 has shown strong elicitation of defense–related gene transcription leading to pathogenesis related (PR) protein production and an increase in the activities of glucanase (GLU), chitinase (CHI), peroxidase (POD) and LOX in Valencia fruit [10]. Iturin A activates the expression of *PR1* and *plant defensin 1*.*2* (*PDF1*.*2*) genes which are important genes in the SA and JA signaling pathways, respectively [9]. Surfactin was shown to be involved in the resistance process by triggering the transcriptional level of *LOX* and *POD* genes [10]. The potential of *Bacillus* CLPs to trigger ISR in part, at least, by reinforcing basal plant defense responses was shown in rice against *Rhizoctonia solani* [11]. However, the specific proteomes regulated by which these CLPs act in the systemic stimulation pathways of the host plants, relevant to the ISR pathway is still unknown. In citrus research, studies of changes in transcriptional and proteome patterns are powerful approaches to gaining more understanding of signal transduction in host plants during stresses. Proteomics coupled with LC-MS-based technique have been recognized as the potential method to identify and characterize citrus proteins to increase the knowledge of citrus proteomes [12].

The current work focused on the individual effect of *B*. *subtilis* ABS-S14 CLPs (fengycin, iturin A, and surfactin) on the transcriptional changes of defense related genes and proteomic profiling of the CLP treated mandarin fruit tissues in response to stresses with and without *P*. *digitatum* invasion. Moreover, comparative binding interaction of individual CLP to plant derived proteins upon *P*. *digitatum* infection was investigated to determine the specific targeted plant molecules to enable plant resistance in postharvest mandarin fruit. The interaction of CLP-binding proteins offers the crucial role of each CLP in its specific elicitor function in triggering plant defensive capacity of mandarin fruit to stress upon pathogen challenging. These specific stimuli enhanced by the CLPs product of the antagonist *B*. *subtilis* ABS-S14, then are able to subsequently enhance fast defense mechanisms in mandarins to reduce the activity of the green mold pathogen.

## Materials and methods

### Plant material and microorganisms

Mandarin fruit (*Citrus reticulata* Blanco cv. Shogun) of uniform size and maturity without physical injuries or infections were collected from commercial orchards located in Loei province, northeastern Thailand (Latitude: 17.4932964 | Longitude: 101.7177129; Altitude: 242 meters) under organic controlled plantation management conditions and without any postharvest treatment was handled [13]. The artificial wounding on mandarin fruit was followed the method as reported in a published work [2].

The fruit-rot fungal pathogen, *P*. *digitatum* was obtained from partially green mold infected mandarin fruit the isolated strain was identified microbiologically and kept in the Fungal Biodiversity Laboratory, National Center for Genetic Engineering and Biotechnology, National Science and Technology Development Agency, Thailand [13]. A conidial spore suspension was adjusted to 10^7^ spores L^-1^ in sterile distilled water using a haemocytometer. The antagonistic strain *B*. *subtilis* ABS-S14 which was used for crude extract preparation was cultured as detailed in [10].

### Preparation of the individual CLP

A crude extract of *B*. *subtilis* ABS-S14 cell-free culture was prepared from 50 g L^-1^ stock solution in 80% ethanol and its antifungal activity was tested using a disc diffusion assay [14]. Fractions of fengycin, iturin A, and surfactin were recovered by ethanol extraction and prepared for PTLC using silica gel according to the previous method [10]. To increase the purity of the CLP fractions, each fraction was loaded into a C18 solid phase extraction (SPE) cartridge (Sep-Pak, Vac 12cc (2 g), silica, 15–105 μm, 125 Å pore size, Waters, USA). A sorbent matrix was eluted by step gradients of acetonitrile (ACN) in 0.1% trifluoroacetic acid (TFA) in order to isolate fengycin, iturin A, and surfactin at concentrations of from 40% to 55%, 25% to 35%, and 60% to 80%, respectively. All the eluting fractions were dried using a rotary evaporator and re-dissolved in 80% ethanol. The antifungal activity of the individual CLP was tested by half maximal efficient concentration (EC_50_) assay [2]. Identification of the CLPs was performed using HPLC and MALDI-TOF MS techniques following a previous study [10]. Purified fractions of fengycin, iturin A, and surfactin were identified using retention time of RP-HPLC and *m/z* ratio of MALDI-TOF MS analysis comparing to their commercial standards (Sigma-Aldrich, USA) as described in a previous report [10].

### Preparation of fruit treatments for gene expression and proteomic profiling analysis

This experiment was designed to compare (a) the single effect of individual CLP and (b) CLP following with *P*. *digitatum* co-application on the transcriptional changes in the targeted genes on wounded sites of mandarin fruit. Artificial wounds were made on the mandarin peel (flavedo) following previously reported methods [10]. In the first treatment group, aliquots of 20 μL each of fengycin, iturin A, and surfactin solution were dropped onto the wound sites at the same concentration (1 g L^-1^). The second treatment group was conducted in the same manner as the first set and, it was followed by fungal inoculation within two hours with an aliquot of 20 μL of 10^7^ spores L^-1^ of *P*. *digitatum* conidial suspension. Sterile distilled water was used as a control [10, 13]. All the treatments consisted of five fruit with three replicates and were kept in sealed plastic boxes containing a cup of water to maintain high humidity at 25 °C for 0, 24, 48, 72 h. The treated flavedo tissues were collected approximately 1 cm away from the wound site [10, 13]. They were pooled and ground into fine powder using liquid nitrogen just prior to analysis.

### Preparation of fruit treatments for CLP-protein binding determination

This experiment was conducted to investigate the flavedo proteins that bound to individual CLP with saturated manner in C18 pipette tip columns. The protein extracts collected from fungal and non-fungal treatments of mandarin fruit tissues were prepared. Equal volumes of 20 μL of the conidial suspension of *P*. *digitatum* (10^7^ spores L^-1^) or sterile distilled water (control) were dropped onto wound sites of the fruit in each treatment group. All the treatments consisted of five fruit with three replicates and were incubated at 25 °C for 48 h. The proteins were extracted from the collected tissue samples as previously described [10]. The experiment was repeated twice.

### Transcriptional change study

The total RNA was extracted from the fine powder of the treated flavedo tissue obtained from the experiment described above using TRI Reagent [13, 15]. The RNA concentration and purity were measured by the absorbance at 260/280 nm (NanoDrop, Thermo Scientific, USA). The Improm-II Reverse Transcription System (Promega) was used to reverse-transcribe the DNase I-treated total RNA to cDNA [15]. Quantitative real time (qRT)-PCR (*Exicycler* 96, Bioneer, Republic of Korea) was conducted [13, 16] (40 cycles of amplification; denature template at 95 °C for 30 sec, anneal primer at 60 °C for 30 sec, extension at 72 °C for 30 sec) [10, 13] with a KAPA SYBR FAST qPCR kit (Kapa Biosystems, USA) to quantify the transcription abundance [13, 16]. Primers for nine targeted genes were designed as listed in S1 Table as described in previous studies; elongation factor 1 alpha (*EF-1a*) (a housekeeping gene), *LOX*, *CHI*, *GLU*, *POD* [10], *PAL* [17], *ACS1*, *ACO* [18] and *PR1* [19]. The internal control was performed by the *EF-1α* transcript [10, 13]. The transcriptional fold changes were determined by (E_target_)^ΔCt, target (calibrator—test)^/(E_ref_)^ΔCt, ref (calibrator—test)^, where E is amplification efficiency and Ct is cycle threshold [20]. The lowest value in the sterile water treatment (control) was defined as the expression level for all genes [10, 13]. The results were expressed as mRNA fold increases over the control sample.

### Protein extraction

The fine-powder samples prepared as described above were prepared in acetone powder form. The total proteins were further extracted by 0.5% sodium dodecyl sulfate (SDS). Sodium deoxycholate (0.15%) and trichloroacetic acid (72%) solution were used for precipitation [21]. Then, the extracted proteins were washed in cold acetone and re-dissolved in 0.5% SDS.

### Proteomic profiling

The extracted proteins in each treatment and a low molecular weight protein standard marker (Amersham Biosciences, UK) were fractionated on 12.5% SDS-PAGE (Atto, Tokyo, Japan). Silver staining of proteins was performed to the gel following the previous method [22]. In-gel digestion was performed on the excised gel pieces (1 mm^3^) according to a previous study [23]. The peptide extractions were pooled and kept at -80 °C for mass spectrometric analysis. The same identified proteins found in sterile distilled water (control) and untreated fruit with artificial wound site were subtracted prior to analyze a comparative study to cut down the effect of sterile distilled water and wound stress, respectively.

### CLP–protein binding assay

**Fig 1:**
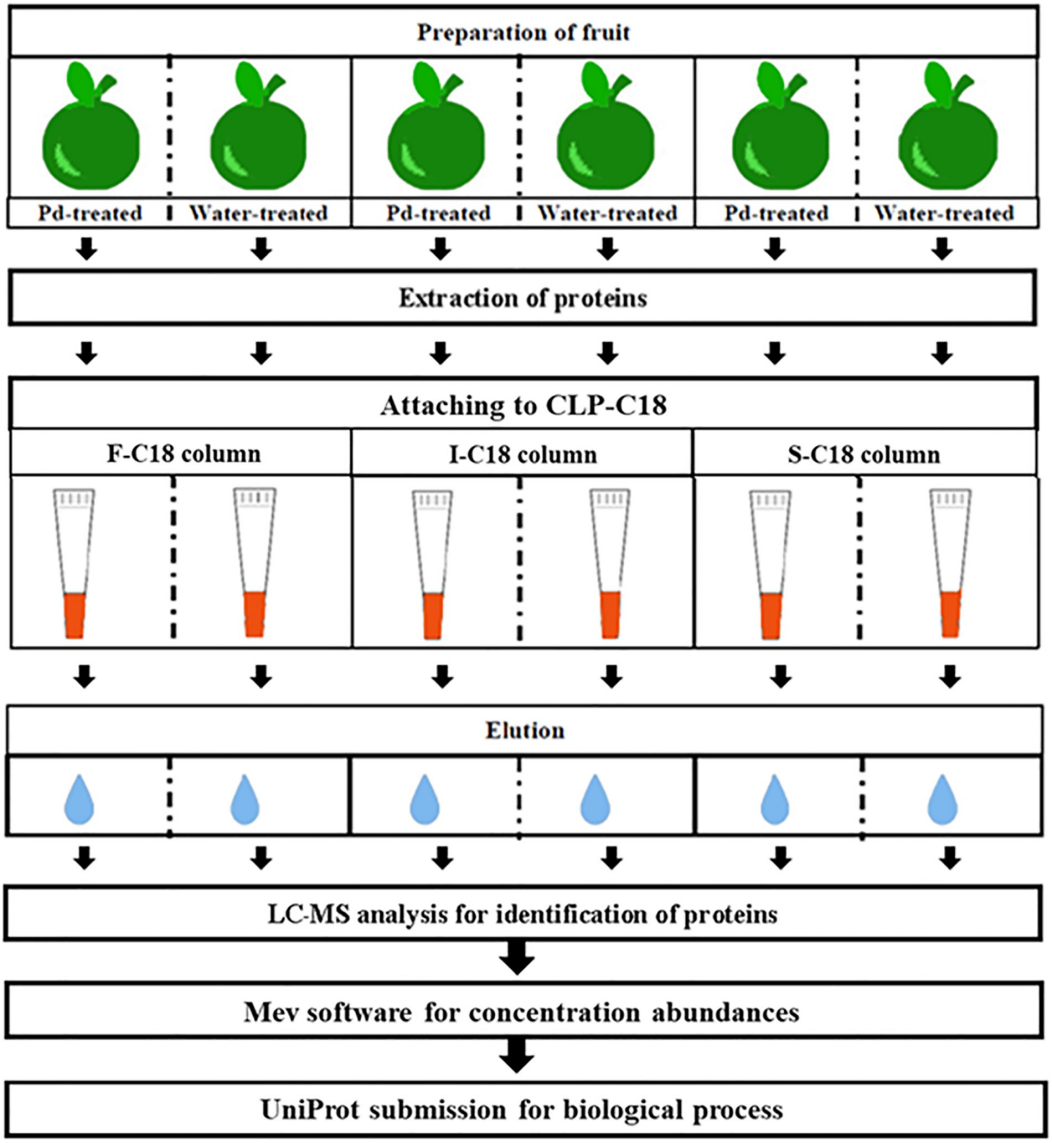
An overview of the protein-CLP binding workflow. Abbreviations: Pd, *Penicillium digitatum*; F, fengycin; I, iturin A; S, surfactin.

This experiment was performed to investigate the binding interaction of the individual CLP with flavedo tissue protein extract obtained after pathogen infection. The interaction between CLP molecules and plant defensive proteins may offer a means of identifying the specific intermediates in host plants that may be involved in the priming process and signal transduction pathways leading to activation of the plant immunity system responding to stresses in mandarin fruit. The flow chart work of CLP-protein binding assay was shown in Fig 1.

The preparation of individual saturated CLP columns was carried out employing C18-pipette tips (Ziptip Pipette Tips 10 μL spherical silica, 15 μm, 200 Å pore size, 31 mm, Merck, Germany) as the SPE columns. The activation of the C18 was performed three times by up-and down pipetting of 100% ACN (10 μL) and 0.1% TFA (10 μL). To ensure that the CLPs were entirely bound to the C18 in a saturated manner, concentrations of 5, 6, and 7 μg CLP solution were applied and eluted with unbound CLP solution. The left-over unbound CLP was monitored using a NanoDrop spectrophotometer for calculation of binding percentage.

A binding experiment using the individual saturated CLP C18-pipette tips was conducted into two paralleled set. The first and the second set were loaded with 5 μg of *P*. *digitatum*-treated protein extracts and sterile distilled water-treated proteins (control), respectively. Each set consisted of three replications and the experiment was repeated twice. The extracted proteins were pipetted up and down three times in C18-pipette tips. The unbound proteins were removed from the C18-pipette tips by the same means with a micropipette, using ACN in a 0.1% TFA solution with concentrations of 40%, 25%, and 65% for the fengycin, iturin A, and surfactin saturated C18-pipette, respectively. Finally, 100% ACN was applied in the C18-pipette tips to elute all the attached proteins for the collection of bound proteins.

To monitor the specific binding ability of extracted proteins that obtained from *P*. *digitatum* and sterile distilled water-treated flavedo, with CLP in the C18-pipette tips, those protein extracts were loaded into C18-pipette tips by pipetting up and down three times. The unbound proteins were removed from the C18-pipette tips by the same means with a micropipette. The solution of 100% ACN was applied in the C18-pipette tips to elute all the attached proteins which bound to C18 molecules in the pipette tip. The bound proteins which were found in both CLP C18-pipette tips and C18-pipette tips without CLP binding were subtracted to determine the binding ability of extracted proteins to CLP molecules.

The bound protein eluting solution derived from the same replicates and treatments was pooled, dried, and re-dissolved in 0.02 N ammonium bicarbonate. Aliquots of 5 μL of 0.02 N dithiothreitol in 0.02 N ammonium bicarbonate and 20 μL of 0.1 N iodoacetamide in 0.02 N ammonium bicarbonate were added to the bound proteins. Then, a trypsin digestion process was carried out in 10 μL of 0.01 g L^-1^ trypsin solution (Promega, USA) which was incubated overnight prior to use for mass spectrometric analysis.

### LC-MS/MS analysis, quantitation and identification of proteins

An HCTultra PTM Discovery System (Bruker Daltonics Ltd., U.K.) coupled to an UltiMate 3000 LC System (Dionex Ltd., U.K on a nanocolumn (PepSwift monolithic column 100 *μ*m i.d. x 50 mm) was used for the analysis of each peptide solution. The running system for LC-MS/MS was conducted following the previous method [23]. DeCyder MS Differential Analysis software (DeCyderMS, GE Healthcare) [24, 25] was used to perform protein quantitation. The converted raw LC-MS data was performed via PepDetect module for automated peptide detection, charge state assignments, and quantitation based on the peptide ion signal intensities in MS mode. A database searching was conducted by submitted analyzed MS/MS data from DeCyderMS to the Mascot software (Matrix Science, London, UK) [26] against the NCBI database (Viridiplantae for proteomic profiling including Viridiplantae and Fungi for CLP-protein association). Data normalization and the quantification of the changes in protein abundance were performed between the control and treated samples and presented via *Multi Figure Experiment Viewer* (Mev) software version 4.6.1 in the form of the heatmap [27]. The list comparison of identified proteins were displayed in Venn diagrams [28]. The proteins were submitted to UniProt (https://www.uniprot.org/) for biological processing [29].

### Statistical analysis

The data sets were analyzed for significant differences by ANOVA. Differences at *p* ≤ 0.05 were considered to be significant. Tukey’s range test was used to establish the significant differences in mean values which were reported as the mean ± standard error (n = 3).

## Results

### Gene expression relating to plant hormone signaling pathways

**Fig 2:**
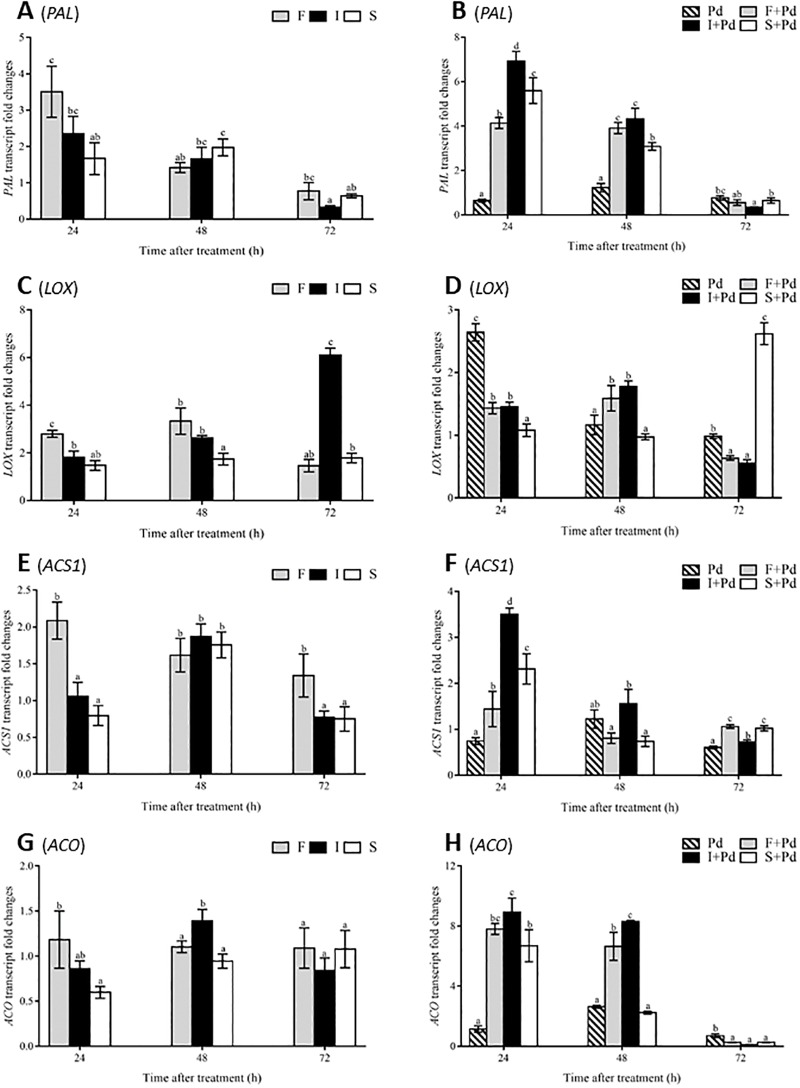
Transcriptional fold changes relating to plant hormone signaling pathways by CLP induction in mandarin fruit. Transcript abundances without fungal infection; (A), (C), (E) and (G) and with fungal infection; (B), (D), (F) and (H) of (A), (B) PAL, (C), (D) LOX, (E), (F) ACS1 and (G), (H) ACO genes. Vertical bars represent standard errors of the mean value of three trials; columns with the same letter above them show no significant difference to each other at p ≤ 0.05 according to the Tukey’s range test. Abbreviations: F, fengycin; I, iturin A; S, surfactin; Pd, *Penicillium digitatum*; F+Pd, fengycin co-applied with *P*. *digitatum*; I+Pd, iturin A co-applied with *P*. *digitatum*; S+Pd, surfactin co-applied with *P*. *digitatum*.

Induction of the expression of *PAL*, *LOX*, *ACS1* and *ACO*, key genes involved in the SA (*PAL*), JA (*LOX)*, and ET (*ACS1* and *ACO*) signaling pathways, by individual CLP with and without co-application of *P*. *digitatum* was conducted. In the fengycin treatment the maximum gene expression of the *PAL* (3.5-fold) was induced at 24 h post-treatment (Fig 2A). During co-application with fungal infection, the pathogen itself had only a small effect on all the gene expressions tested except in *LOX* gene expression (Fig 2B, 2D, 2F and 2H). Iturin A triggered the highest amount of *PAL* transcripts (6.9-fold) at 24 h post-treatment (Fig 2B). The abundance of *PAL* transcripts decreased continually from 24 to 72 h post-treatment in all the CLP treatments (Fig 2A and 2B). Iturin A showed the greatest induction of *LOX* transcripts (6-fold) at 72 h post-treatment without fungal attack (Fig 2C). In green mold infected fruit, *P*. *digitatum* elicited the highest level of *LOX* gene expression (2.6-fold) as early as 24 h post inoculation (Fig 2D), while the same activity was detected later in surfactin-treated tissues at 72 h post treatment. In Fig 2E, fengycin presented the highest effect on the *ACS1* transcript level at 24 h post-treatment (2.1-fold) but this gradually declined towards 72 h post-treatment. However, iturin A elicited the highest *ACS1* transcription (3.5-fold) at 24 h post-treatment which was then decreased at 48 and 72 h post-treatment during green mold attack (Fig 2F). In fengycin the treated tissues revealed a constant level of *ACO* transcripts without fungal infection, but there was a non-significant difference of *ACO* expression by other CLPs at later treatment times (Fig 2G). It was noticeable that iturin A showed the strongest elicitation of *ACO* at 24 h post-treatment (8.9-fold) with only a small decline at 48 h post-treatment (8.3-fold) during fungal infection but a lesser effect on the elicitation of *ACO* expression was detected in the fengycin-treated tissues (Fig 2H).

### PR protein gene expression

**Fig 3:**
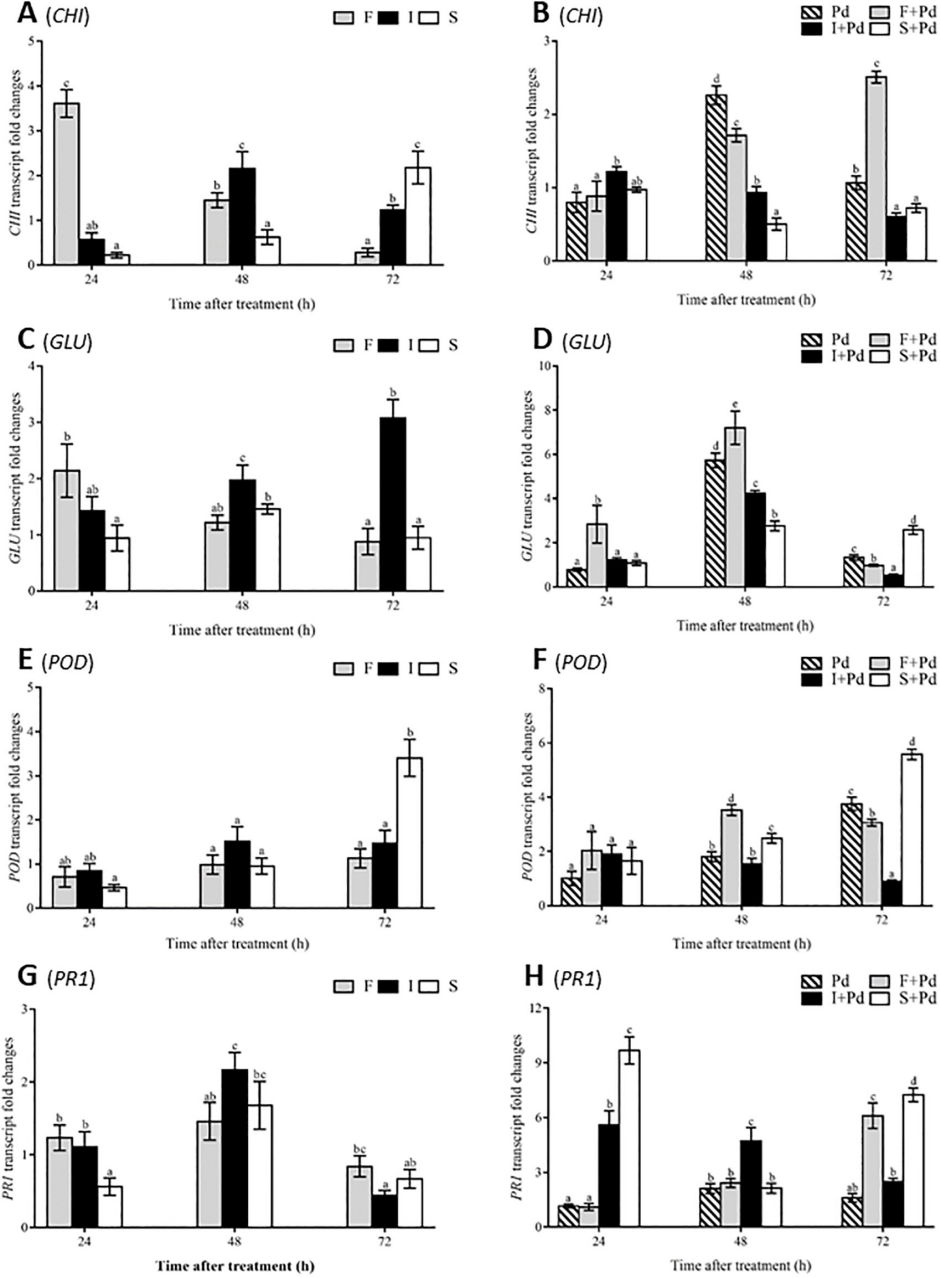
Transcriptional fold changes relating to PR proteins induced by CLPs in mandarin fruit. Transcript abundances without fungal infection; (A), (C), (E) and (G) and with fungal infection; (B), (D), (F) and (H) of (A), (B) *CHI*, (C), (D) *GLU*, (E), (F) *POD* and (G), (H) *PR1* genes. Vertical bars represent standard errors of the mean value of three trials; columns with the same letter above them show no significant difference to each other at *p* ≤ 0.05 according to the Tukey’s range test. Abbreviations: F, fengycin; I, iturin A; S, surfactin; Pd, *Penicillium digitatum*; F+Pd, fengycin co-applied with *P*. *digitatum*; I+Pd, iturin A co-applied with *P*. *digitatum*; S+Pd, surfactin co-applied with *P*. *digitatum*.

To confirm the effects of individual CLP on eliciting plant PR protein production, a comparative study of the changes in transcription patterns of *CHI*, *GLU*, *POD*, and *PR1* in flavedo tissues treated with CLPs, with and without following pathogen attack was conducted. The level of induction of *CHI* transcripts reached a maximum in the fengycin treatment within 24 h post-treatment and thereafter continually decreased, whereas an increase in the *CHI* transcript level was detected in surfactin-treated fruit between 24 and 72 h post-treatment (Fig 3A). Following infection with *P*. *digitatum*, the fengycin treatment showed a marked increase in the induction of the *CHI* gene at 48 h post-treatment (2.3-fold) and 72 h post-treatment (2.5-fold) (Fig 3B). Moreover, fengycin produced an increasing trend of *CHI* transcripts from 24 to 72 h post-treatment, which was opposite to the decreasing trend detected in the iturin A treatment. The expression of the *GLU* gene displayed a decreasing trend in the fengycin treatment which was the opposite of the trend in the iturin A-treated flavedo without pathogen stress (Fig 3C).

As can be observed in Fig 3D, fengycin co-applied with *P*. *digitatum* synergistically activated the highest level of *GLU* accumulation (7.2-fold) at 48 h post-treatment. The treated flavedo showed the highest level of *POD* expression at 72 h post-treatment induced by surfactin (3.4-fold) and by surfactin co-applied with *P*. *digitatum* (5.6-fold) (Fig 3E and 3F, respectively). The abundance of *PR1* transcripts reached its maximum when induced by iturin A (2.2-fold) at 48 h post-treatment without fungal attack (Fig 3G). In contrast, surfactin showed a significant effect when co-applied with *P*. *digitatum* on the accumulation of *PR1* transcripts at 24 h post-treatment (9.7-fold) which declined at 72 h post-treatment (7.3-fold) during fungal infection (Fig 3H).

### Plant protein production

**Fig 4:**
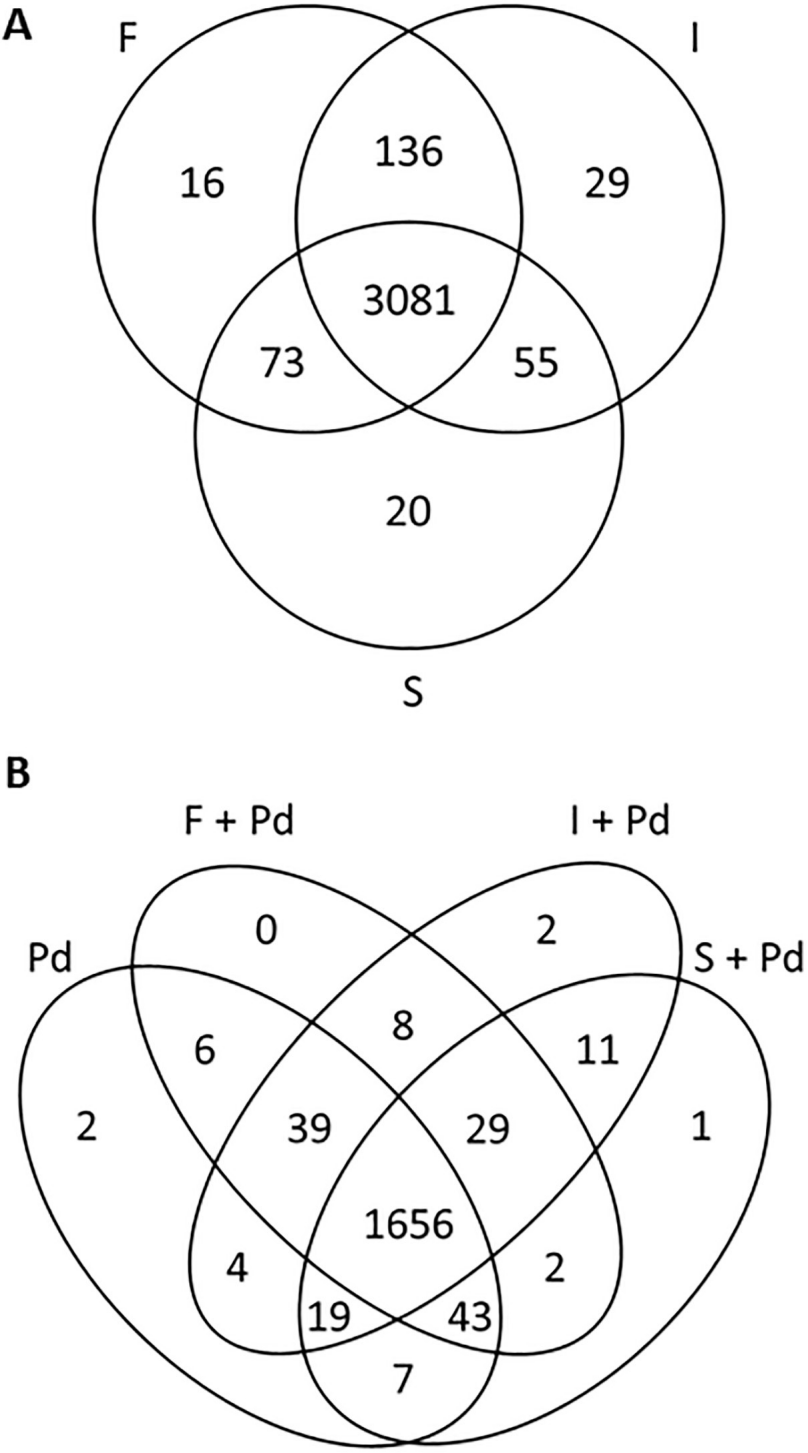
Venn diagram showing the total estimated number of proteins distributed by individual CLP. (A) The three groups of agents responding to stress without fungal infection induced by fengycin (F), iturin A (I), and surfactin (S). (B) The four groups of agents stimulated by *Penicillium digitatum* (Pd), fengycin co-applied with *P*. *digitatum* (F+Pd), iturin A co-applied with *P*. *digitatum* (I+Pd) and surfactin co-applied with *P*. *digitatum* (S+Pd).

**Fig 5:**
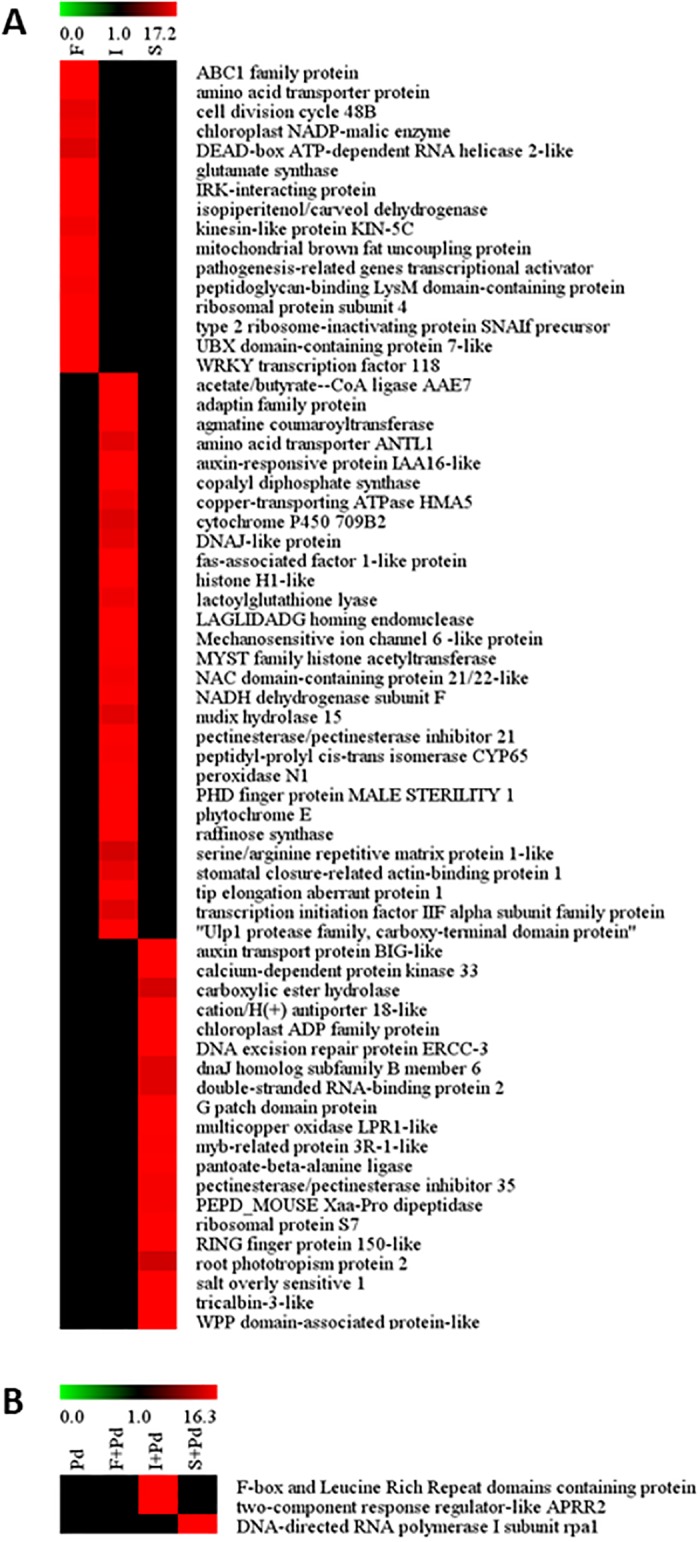
Heatmap showing the relative amount of each protein activated by individual CLP. (A) The three groups of agents responding to stress without fungal infection induced by fengycin (F), iturin A (I), and surfactin (S). (B) The four groups of agents stimulated by *Penicillium digitatum* (Pd), fengycin co-applied with *P*. *digitatum* (F+Pd), iturin A co-applied with *P*. *digitatum* (I+Pd) and surfactin co-applied with *P*. *digitatum* (S+Pd). A color bar of green to red color stands for the intensity of the total ion chromatogram from 0.0 to 17.2 in (A) and 0.0 to 16.3 in (B).

*B*. *subtilis* CLPs induced distinct patterns of plant defensive proteins under stresses with and without fungal infection. Fig 4A shows the 3,081 common proteins which were found in the flavedo tissues treated with fengycin, iturin A, and surfactin without *P*. *digitatum* infection. Fengycin and iturin A produced a common effect in inducing 136 proteins in the flavedo tissues while 73 and 55 proteins were observed in common between the treatments of fengycin and surfactin, and the treatments of iturin A and surfactin, respectively. There were 16, 29, and 20 proteins uniquely produced by the fengycin-, iturin A-, and surfactin-treated groups, respectively. During green mold attack, a large number (1,656) of proteins in host plants were determined in common (Fig 4B) while only eight proteins were detected in common in the fengycin- and iturin A co-applied with *P*. *digitatum* treatments. There were only two common proteins found in the treatments of fengycin and surfactin co-applied with *P*. *digitatum*. The tissues treated with iturin A and surfactin shared 11 identified proteins when responding to fungal attack. One and two unique proteins were found in the surfactin and iturin A treatments co-applied with *P*. *digitatum*, respectively. While in the fengycin counterpart, no unique protein was found. A list of the proteins with their unique characteristics, and significant roles in biological processes obtained from each treatment, with and without fungal infection are shown in S2–S5 Tables. The relative amount of each protein is presented as a heatmap in Fig 5.

### Specific CLP-binding proteins

To emphasize the role of *B*. *subtilis* CLPs involving in elicitation of defensive system against pathogen infection, mass spectrometry and proteomic approaches were applied to monitor the mediator proteins that are responsible for the plant-microbe interaction. Scans of individual saturated CLP in C18-pipette tips with maximum concentration CLP solutions (5 μg) were manually performed following the data obtained from a NanoDrop spectrophotometer. The maximum binding of the CLPs to the C18 was respectively 5, 6, and 7 μg of iturin A, fengycin, and surfactin, which bound to the C18 at 96%, 95%, and 94% (iturin A), 98%, 97% and 96% (fengycin), and 98%, 97% and 94% (surfactin).

The majority of the commonly expressed proteins presented in the *P*. *digitatum* treated and control groups, which were bound to the saturated CLPs in the C18 columns, are illustrated in Venn diagrams in S1 Fig. To confirm the binding ability of extracted proteins to CLP molecules, the bound proteins which were found in both CLP-C18-pipette tips and C18-pipette tips were subtracted from the expressed proteins in S1 Fig. Fengycin captured 81 and 82 proteins in the treated and control groups, respectively as shown in the Venn diagram in S1A Fig. Of those, 78 were entrapped in the saturated C18-pipette tips for both groups while three proteins presented only in the fungal-treated group and four proteins presented only in the control group. For the iturin A-saturated C18–protein interactions, 82 differentially expressed proteins out of a total identified of 85 which were common to the pathogen treated and control distilled water groups were shown in S1B Fig. It was noticeable that only iturin A was able to capture the three unique proteins expressed in the fungal-treated group. Whereas the surfactin trapped only one protein found in the control group out of the common 83 proteins detected in the treatments with and without fungal infection as shown in S1C Fig.

**Fig 6:**
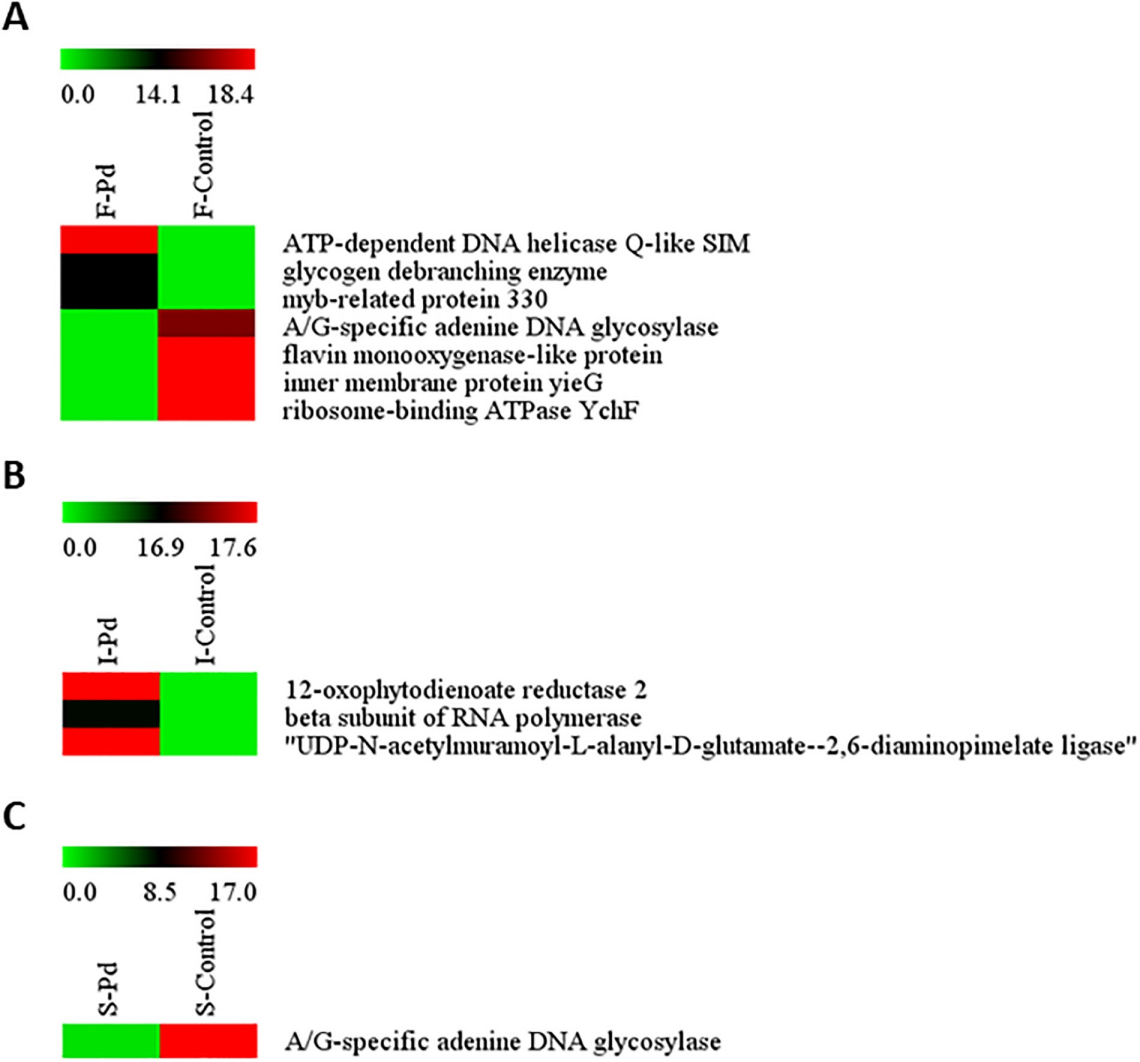
Heatmap showing the relative amount of each protein in individual CLP-protein association. Groups of (A) fengycin-attaching proteins induced by *Penicillium digitatum* (F-Pd) and sterile distilled water (F-Control), (B) iturin A-attaching proteins induced by *P*. *digitatum* (I-Pd) and sterile distilled water (I-Control), (C) surfactin-attaching proteins induced by *P*. *digitatum* (S-Pd) and sterile distilled water (S-Control). A color bar of green to red color stands for the intensity of the total ion chromatogram from 0.0 to 18.4 in (A), 0.0 to 17.6 in (B), and 0.0 to 17.0 in (C).

Identification of the proteins attached to each CLP of *B*. *subtilis* detected in the treatments with and without fungal invasion were determined. Only a small number of unique proteins were found in specific protein-CLP binding groups. Fig 6A illustrates the three unique proteins characterized in fungal-treated hosts which interacted with fengycin and were identified as follows; 1) ATP-dependent DNA helicase Q-like SIM, 2) glycogen debranching enzyme, and 3) myb-related protein 330 occurring in the green mold attack condition. In contrast, there were four unique proteins able to bind with fengycin molecules in non-fungal invasive treatment, which were characterized as 1) A/G-specific adenine DNA glycosylase, 2) flavin monooxygenase-like protein, 3) inner membrane protein yieG, and 4) ribosome-binding ATPase YchF (Fig 6A). Interestingly, in the fungal-treated protein extract, the three unique proteins bound to iturin A were determined to be 1) 12-oxophytodienoate reductase 2, 2) beta’ subunit of RNA polymerase, and 3) UDP-N-acetylmuramoyl-L-alanyl-D-glutamate—2,6-diaminopimelate ligase (Fig 6B). In addition, A/G-specific adenine DNA glycosylase was the only unique protein attached to *B*. *subtilis* surfactin without fungal invasion (Fig 6C). The biological processes of the unique proteins binding to molecules of CLPs are clearly displayed in Table 1. The relative amount of each protein is also shown as a heatmap (Fig 6). The details of all the biologically active proteins are shown in S6 Table.

**Table 1:** List of unique proteins attached to CLP-C18 in the treatments co-applied with and without fungal infection.

No.	Protein name	Accession No.*	Condition	Biological process
1	ATP-dependent DNA helicase Q-like SIM	XP_004960267.1	fengycin-Pd	DNA repair
2	glycogen debranching enzyme	XP_013432022.1	fengycin -Pd	glycogen catabolic process
3	MYB-related protein 330	XP_013659104.1	fengycin-Pd	transcription
4	A/G-specific adenine DNA glycosylase	XP_013632165.1	fengycin and surfactin	base-excision repair
5	flavin monooxygenase-like protein	OAA54389.1	fengycin	oxidation-reduction process
6	inner membrane protein yieG	XP_003176899.1	fengycin	transport
7	ribosome-binding ATPase YchF	XP_017602863.1	fengycin	response to oxidative stress
8	12-oxophytodienoate reductase 2	XP_015643914.1	iturin A-Pd	oxylipin biosynthetic process
9	beta subunit of RNA polymerase	YP_002000409.1	iturin A-Pd	transcription
10	UDP-N-acetylmuramoyl-L-alanyl-D-glutamate--2,6-diaminopimelate ligase	JAT63352.1	iturin A-Pd	cell wall organization

*Penicillium digitatum*, Pd.

*The accession numbers can be obtained from NCBI database.

## Discussion

Non-pathogenic rhizobacteria such as *Bacillus* species which produce CLPs, are powerful in stimulating plant immunity through ISR system in a particular of citrus biocontrol [3, 10, 13, 30]. The mode of CLP actions stimulating plant immunity in postharvest citrus fruit was elucidated by the transcriptional changes [10]. However, the comparative effect of fengycin, iturin A, and surfactin in proteomic response to stress to trigger plant resistance in postharvest mandarin fruit is still unknown. In this study, the mechanisms of fengycin, iturin A, and surfactin initiating the distinguished expression of the defensive genes and proteomic profiling including individual CLP-plant defensive protein interaction in response to stresses were established.

Mandarin fruit treatments with fengycin and iturin A significantly enhanced the levels of *PAL* gene expression at the earliest time post-treatment both with and without fungal infection. This was similar to those achieved in a previous study of PAL enzyme activity elicited by *Bacillus* CLPs in infected mandarin fruit [31]. The *PAL* gene plays a crucial role not only in SA biosynthesis, but also in the phenylpropanoid pathway which produces lignins, flavanoids, and coumarins as weapons against pathogens [32]. In contrast, iturin A showed better ability to amplify the level of *LOX* transcripts than other CLPs in non-fungal treatments. An invasion of green mold in mandarin flavedo increased the expression of the *LOX* gene at an early stage which was equal to that found in the surfactin treatment at a later time point. The action of surfactin in this study was the same manner as a published work [10]. The accumulation of JA is dependent on the expression of the *LOX* gene resulting in ISR activation [5]. Fengycin and iturin A possess a high potential to trigger the *ACS1* and *ACO* production, even without green mold attack, at 24 and 48 h post-treatment, respectively. Following fungal infection, iturin A was the best agent in triggering *ACS1* and *ACO* transcriptions. These results agreed with a previous work of the *ACS1* transcription level elicited by fengycin and the iturin family in the ET signaling pathway in rice [11]. *ACS* and *ACO* gene expression are required for ET biosynthesis resulting in ISR induction [6]. Therefore, this work demonstrates that fengycin, iturin A and surfactin stimulated the immune system in mandarin fruit via JA/ET signaling pathways leading to activation of *PR* gene expression as shown in previous reports [3, 9, 33].

It is known that an increase in PR protein production is one of a host plant’s strategies to protect itself from pathogen diseases [33]. The present investigation of the action of *Bacillus* individual CLPs on inducing gene expression of PR proteins like *CHI*, *GLU*, *POD*, and *PR1* in postharvest mandarin fruit was conducted by qPCR. Fengycin, iturin A, and surfactin showed the potential to stimulate the accumulation of transcripts of *CHI* at 24, 48, and 72 h post treatment in non-fungal infection treatments. Interestingly, only in the fengycin treatment increase of the expression of *CHI* gene following fungal invasion was observed. This trend was similarly observed in the iturin A treatment on the induction of *GLU* gene expression in non-fungal treatment although the greatest accumulation of *GLU* transcript abundance with co- application with fungal infection was found in the fengycin treatment. Surfactin enhanced *POD* expression during with and without fungal attack. *PR1* abundance reached its highest level at 48 h post-treatment in the flavedo tissues treated with iturin A, both with and without green mold infection, while surfactin co-applied with *P*. *digitatum* triggered the greatest level of *PR1* transcription at 24 and 72 h post-treatment. These results correspond to previous works that reported the effect of fengycin, iturin A, and surfactin on the activation of *CHI*, *GLU*, and *POD* in mandarin fruit with fungal infection [10], as well as the enhancement of the *PR1* gene by iturin A elicitation in *Arabidopsis* plants [9]. Therefore, *B*. *subtlis* ABS-S14 CLPs are potent agents promoting *PR* gene expression which leads to increase in PR protein accumulation with antimicrobial and antifungal properties for plant resistance in postharvest mandarin fruit.

Transcriptomes and proteomes are potential approaches to highlight the changes in citrus fruit during development stage [34]. Transcriptional data combined with proteomic data could fulfill the understanding of the individual CLP action activating defensive mechanism in mandarin fruit. In the present study CLPs were found to induce the flavedo tissues of mandarin fruit to synthesize various plant defensive proteins which are associated with many significant pathways of plant immunity under stresses with and without *P*. *digitatum* invasion. This work focused on the functionally active proteins which are unique proteins presented in the fengycin, iturin A and surfactin treatments which are associated with significant biological processes in plant defense during stresses with and without green mold infection. The 16 proteins present in the fengycin treated flavedo’s response to stress without fungal infection are unique proteins showed the important biological processes involving in plant defense [29]. ABC1 family protein was one of 16 unique proteins in fengycin treatment without fungal invasion which is involved in plant development and supports secondary metabolite accumulation for plant defense [35], and also functions in the ubiquinone biosynthetic process [29]. Ubiquinone plays a crucial role relating to reactive oxygen species (ROS) in stress responses [36]. However, no unique protein appeared in the fengycin treatment during fungal infection.

In the iturin A treatment, distinct patterns of proteins in the flavedo tissues were revealed both with and without fungal attack. The 29 unique proteins presented in the iturin A treated tissues are known to play a role in a plant’s response to stress without green mold infection. Auxin-responsive protein IAA16-like and NAC domain-containing protein 21/22-like were 2 of 29 unique proteins in the iturin A treatment without green mold infection which functions in the auxin-activated signaling pathway [29]. Auxin is a phytohormone in plants acting in plant growth and plant defense. Auxin signaling shares a lot of common components with JA signaling, resulting in plant resistance outcomes via ISR [37]. Noticeably, higher numbers of unique proteins were observed to be activated by iturin A in the treatment without fungal infection than iturin A co-applied with the fungal pathogen. In the treatment of iturin A co-applied with *P*. *digitatum*, two unique proteins were stimulated, F-box and leucine rich repeat domains containing protein, which is related to the ubiquitin-dependent protein catabolic process [29]. Ubiquitin is a vital component in the ubiquitin pathway which is instrumental in responding to stress resulting in the defense mechanism [38]. A second protein was two-component response regulator-like APRR2 which functions in the phosphorelay signal transduction system [29]. Phosphorelays have been reported to be implicated in cytokinin regulation and signal responses for higher plants in response to bacteria [39].

Without *P*. *digitatum* infection of surfactin-treated flavedo tissues, auxin transport protein BIG-like was one of 20 unique proteins involved in the biological processes relating to auxin-activated signaling pathway [29, 37]. Furthermore, DNA-directed RNA polymerase I subunit rpa1 was the only unique protein detected in the surfactin treatment co-applied with pathogen, which functions in the negative regulation of protein localization to the nucleolus [29]. Therefore, CLPs alone were able to trigger unique proteins, which functioned in defensive systems such as plant hormone transductions and ROS metabolism to promote the plant defense response during stresses with and without fungal infection as reported the biological process in a previous work [11]. Markedly, iturin A treatment increased the higher number of proteins relating to ISR activation for plant resistance than fengycin and surfactin did in response to stress without fungal infection.

To determine the intermediate signals triggering plant immunity during stresses, CLP-protein binding assay coupled with LC-MS/MS can explain the binding ability of CLPs to targeted proteins involving in elicitation of host plant resistance. In this study, the distinguished binding potentiality of fengycin, iturin A, and surfactin to the flavedo-extracted proteins obtained under the conditions with and without green mold invasion was demonstrated. To indicate the specific role of individual CLP on activation of plant resistance, the unique proteins with defensive function were highly focused.

During fungal attack, fengycin had the ability to attach three unique proteins, which were; 1) ATP-dependent DNA helicase Q-like SIM; 2) glycogen debranching enzyme; and 3) MYB-related protein 330, relating to DNA repair, glycogen catabolic process, and transcription, respectively, through biological processes, [29]. The four unique proteins which showed up in the stress without pathogen infection attaching to fengycin were (1) A/G-specific adenine DNA glycosylase; (2) flavin monooxygenase-like protein; (3) inner membrane protein yieG; and (4) ribosome-binding ATPase YchF, which have functions in base-excision repair, the oxidation-reduction process, transport, and the response to oxidative stress, respectively, through biological processes [29]. One of the vital effects of fungal attack is DNA damage. Fengycin is able to trigger the proteins implicated in the DNA repair process, which is involved in the repair of lesions in DNA for specialized proteins and regulatory pathways [40].

Surprisingly, no unique binding protein was found in the non-fungal infected iturin A treatment. However, the three unique proteins attaching to iturin A following *P*. *digitatum* invasion were characterized as (1) 12-oxophytodienoate reductase 2; (2) beta subunit of RNA polymerase; and (3) UDP-N-acetylmuramoyl-L-alanyl-D-glutamate—2,6-diaminopimelate ligase, which are associated with the oxylipin biosynthetic process, transcription, and cell wall organization, respectively, through biological processes [29]. The oxylipin biosynthetic process produces an accumulation of JA, which plays a vital role in the JA signaling pathway for the elicitation of plant immunity [5] and is effective in the production of PR proteins [33].

In addition, without fungal attack A/G-specific adenine DNA glycosylase was the only unique plant protein attaching to surfactin. This protein has been demonstrated to function in the base-excision repair process [29, 41]. The distinct mechanism of individual CLP attaching to unique proteins for plant resistance, therefore, may depend on the type of stress condition.

**Fig 7:**
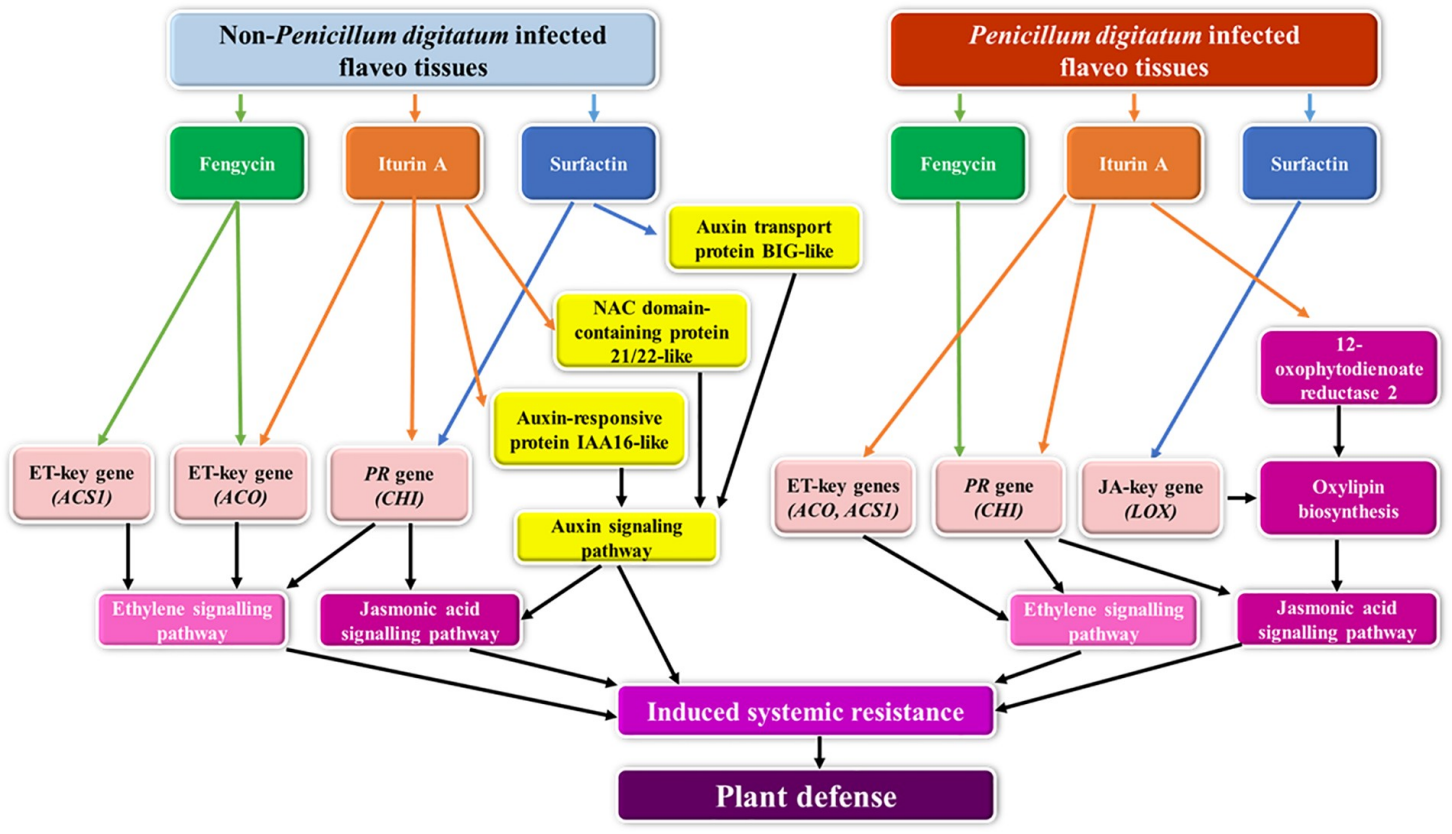
Proposed model of CLPs to induce ISR pathway in postharvest mandarin fruit. Without *Penicillium digitatum* infection, fengycin induced the expression of key genes in ethylene (ET) signaling pathway. Iturin A could induce the ET key gene and *chitinase* (*CHI*) gene (jasmonic acid, JA/ET responsive gene) and the proteins; auxin-responsive protein IAA16-like and NAC domain-containing protein 21/22-like functioning in auxin signaling pathway. Surfactin also elicited the expression of *CHI* gene and a protein involves in auxin signaling pathway resulting in activation of induced systemic resistance (ISR) for plant resistance. In *P*. *digitatum* infected treatments, fengycin and surfactin showed ability to trigger *CHI* gene and *lipoxygenase* (*LOX*) genes, respectively. While iturin A stimulated the expression of ET key genes and *CHI* genes. Moreover, iturin A could attached to 12-oxophytodienoate reductase 2 which is involved in the oxylipin biosynthetic process resulting in ISR activation via JA signaling pathway for plant resistance. (Arrows in green, responding to fengycin treatment; orange, responding to iturin A treatment; blue, responding to surfactin treatment; black, activating ISR pathway and plant defense).

Overall, fengycin may play a role to enhance plant defensive-protein production during stresses with and without fungal infection by connecting to the proteins involved in the transcription process. Similarly, surfactin operated through an equivalent mechanism in the reaction to stress without fungal infection, while iturin A prefers to stimulate plant immunity via the JA-mediating signaling pathway for ISR activation against green mold infection, a different mechanism to that operating for fengycin and surfactin in the stress response. In summary, fengycin, iturin A, and surfactin have different potentials to trigger defensive genes expression of key genes in the plant hormone modulating system and specific *PR* genes leading to plant resistance at transcriptional level. At proteomic level, iturin A showed the stronger power to increase the production of unique proteins involving ISR mechanism comparing with fengycin and surfactin. Moreover, CLP-protein binding assay coupled with proteomic approach revealed the binding ability of iturin A to the intermediate signal molecules in ISR pathway against pathogen infection. Ability of iturin A in activating ISR mechanism was the same manner as the previous report [9]. The proposed mechanisms of each *Bacillus* CLP activate plant resistance through ISR pathway was shown in Fig 7. It could be concluded that CLPs enhances the plant resistance via plant hormone signaling pathways, especially, ISR mechanism in response to stress following with and without *P*. *digitatum* infection in postharvest mandarin fruit.

## Conclusions

The current study revealed the specific mechanism at transcriptional and proteomic levels of the individual CLP obtained from *B*. *subtilis* ABS-S14 triggering plant immunity in postharvest mandarin fruit. Proteomic approach combined with LC-MS/MS provides the understanding of CLP-protein interaction related to ISR elicitation. This provides a better understanding of the processes by which *B*. *subtilis* CLPs mediate responses in host plants when exposed to stresses. Ultimately, induction of ISR system in mandarin fruit would be provoked pathogen invasion. Future experiments should focus on the cascade effects of on the intermediate chemicals in steps of plant hormone modulating signal transductions by which the plant’s defenses are stimulated. This information may lead to understanding the outcome of plant-microbe interaction under pathogen invasion and enable to wisely manipulate application of *B*. *subtilis* as an effective biological agent in postharvest treatment of fruit.

## Supporting information

S1 Fig Venn diagram showing the total estimated number of proteins distributed in individual CLP-protein association. (A) Treatments of fengycin-attaching proteins induced by *Penicillium digitatum* (F-Pd) and sterile distilled water (F-Control). (B) Treatments of iturin A-attaching proteins induced by *P*. *digitatum* (I-Pd) and sterile distilled water (I-Control). (C) Treatments of surfactin-attaching proteins induced by *P*. *digitatum* (S-Pd) and sterile distilled water (S-Control).
(TIF)

S1 Table Gene-specific primer sequences and amplified products of defense-related and reference genes. (XLSX)

S2 Table List of unique proteins presenting in fengycin treatment without fungal infection. (XLSX)

S3 Table List of unique proteins presenting in iturin A treatment without fungal infection. (XLSX)

S4 Table List of unique proteins presenting in surfactin treatment without fungal infection. (XLSX)

S5 Table List of unique proteins presenting in each treatment during fungal infection. (XLSX)

S6 Table List of unique proteins attaching to CLP-C18 pipette tip. (XLSX)
