# Supplementary figures and images for: Targeted transcriptional and proteomic studies explicate specific roles of Bacillus subtilis iturin A, fengycin, and surfactin on elicitation of defensive systems in mandarin fruit during stress
Source: PLoS One. 2019 May 23;14(5):e0217202. doi: 10.1371/journal.pone.0217202 (PMC6532888; doi:10.1371/journal.pone.0217202)

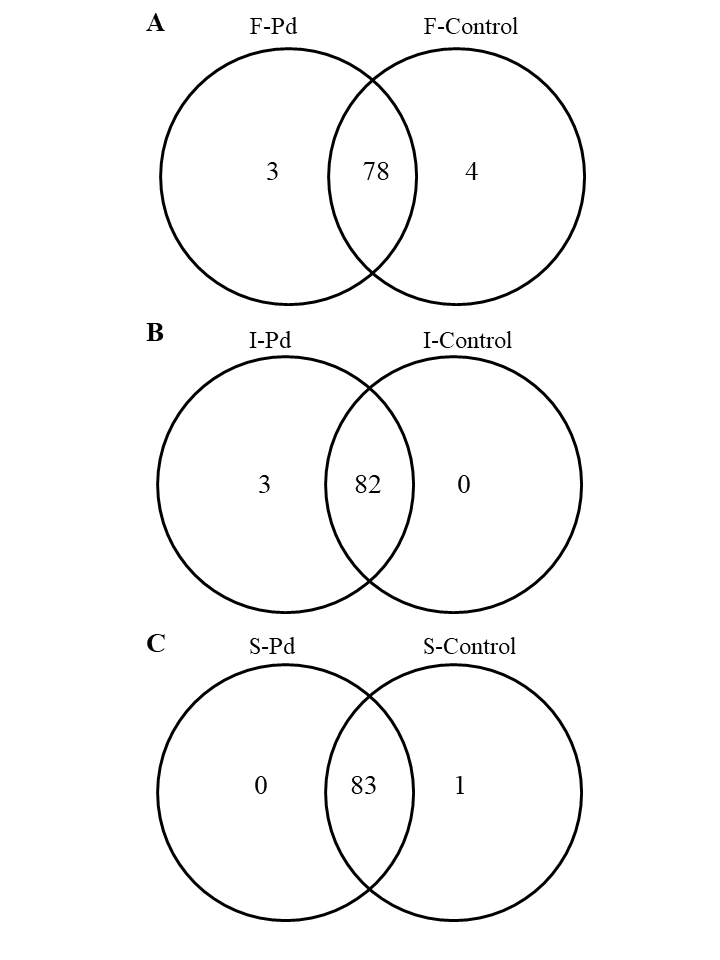

Supplement: S1 Fig — (A) Treatments of fengycin-attaching proteins induced by Penicillium digitatum (F-Pd) and sterile distilled water (F-Control). (B) Treatments of iturin A-attaching proteins induced by P. digitatum (I-Pd) and sterile distilled water (I-Control). (C) Treatments of surfactin-attaching proteins induced by P. digitatum (S-Pd) and sterile distilled water (S-Control). (TIF) [file pone.0217202.s001.tif]
